# Control of Contaminant Transport Caused by Open-Air Heavy Metal Slag in Zhehai, Southwest China

**DOI:** 10.3390/ijerph16030443

**Published:** 2019-02-02

**Authors:** Jiang Zhao, Zhihua Chen, Tao Wang, Caijuan Xiang, Mingming Luo, Hongxin Yuan

**Affiliations:** 1 School of Environmental Studies, China University of Geosciences, 388 Lumo Rd, Wuhan 430074, China; jzhao@cug.edu.cn (J.Z.); zhchen@cug.edu.cn (Z.C.); 13026151960@163.com (C.X.); luomingming@cug.edu.cn (M.L.); 2 Yunan Chihong Zinc and Germanium Co., Ltd. Huize Branch, Huize 654212, China; hxyuan_ch@163.com

**Keywords:** open-air heavy metal, groundwater modeling, reactive transport, in situ treatment

## Abstract

Slag heaps are formed by mining waste materials, and the improper treatment of leachate from such heaps can threaten nearby aquifers. The Zhehai slag heap in Yunnan Province, China, contains 2.7 million tons of zinc and cadmium slag, and is considered a heavy metal source threatening the local groundwater safety, however, the severity of contamination remains unknown. In this study, numerical modeling was used to predict the groundwater flow and contaminant transport in this area based on field data. The results show that the atmospheric precipitation infiltration recharge at the top of the heap is 81.8 m^3^/d, accounting for 93.76% of total infiltration. The south and east sides of the area are the main outflow channels for contaminants, accounting for 93.25% of the total discharge around the heap. To reduce aquifer contamination, an in situ system involving a “controlling the source, ‘breaking’ the path, and intercepting the flow” (CSBPIF) strategy is established. The results indicate that the system performs well because it not only decreases the flow velocity but also reduces the concentrations of contaminants adsorbed by clay media. Moreover, the equivalent bottom liner thicknesses of the clay layers were calculated to improve the applicability of the CSBPIF system. Compared with ex situ disposal, this scheme provides an economic and effective solution and can be used to prevent and control groundwater pollution in China.

## 1. Introduction

In industrialized areas, human activities influence local ecosystems by constantly producing products such as smelting slag. Slag has led to heavy metal pollution (such as Cd, Cu, Pb, and Zn pollution) in groundwater adjacent to the nickel-copper smelting industry in the central Barents Region [1]. Additionally, arsenic waste from a gold and silver extraction area in Baja California Sur, Mexico [2], and a century-old nonferrous metal mining and smelting area in southern Hunan Province, China [3], has led to severe pollution. China is rapidly becoming one of the largest groundwater and soil remediation regions in the world [4]. Therefore, it is beneficial and necessary to control and remediate contaminated groundwater in China.

In some industrialized areas, metal smelter residue is exposed to air for a long period of time. In addition, precipitation can generate heavy metal leachate from slag heaps. Such leachate poses the most significant risk to the groundwater environment, especially in the absence of a remediation, restriction, or isolation system [5,6,7]. Once the leachate reaches the groundwater table, it is transported laterally with the groundwater and discharged via seepage at the terrain surface or migrates vertically and moves into the deep groundwater [8,9]. If the leachate of heavy metals enters the ecosystem and food chain, it can have harmful effects on human health. Therefore, controlling and remediating metal smelter residues are immense challenges that have attracted global attention [10,11,12,13,14,15].

Ex situ disposal and in situ natural attenuation are the major effective remediation strategies [16]. Excavation followed by ex situ disposal, often known as dig and haul, is a proven and easily implementable remediate method that is applicable to all contaminants. Prior to 1984, excavation and ex situ disposal were the most common methods of remediating hazardous waste sites in the U.S. However, in addition to the cost of transportation, the required technology can be more expensive than that required by other methods when offsite treatment and disposal are included [17]. Moreover, the depth and composition of the materials to be excavated must be considered, and the remediation technology is typically restricted to shallow soils less than approximately 3 m below the ground surface [18]. Soil excavation and transportation can substantially increase the cost and duration of a remediation project. The feasibility and availability of offsite disposal are thus restricted [19,20].

In situ natural attenuation, which involves natural physical, chemical, and biological processes, is a remedial solution that can reduce the concentrations of contaminants in the subsurface. This approach has several advantages, such as the ability to combine multiple remediation technologies [21], a low cost of remediation [22], and few remediation waste products [23]. However, in situ natural attenuation strategies must efficiently prevent and control contaminant migration, and the effects of prevention and control measures on groundwater must be accurately predicted and analyzed [24]. Numerical models are commonly used to solve such problems by rapidly predicting future changes in hydrogeological systems [25].

The slag heap in Zhehai town, Yunnan Province, China, is a major pollution hazard. The heap is approximately 1.0 × 10^7^ m^2^ in area, 3.0 × 10^7^ m^3^ in volume and 4.2 × 10^7^ tons in quantity. It consists of approximately 2.7 × 10^7^ tons of smelting slag produced by the Zhehai smeltery, which began pyro-refining in 1965, 0.5 × 10^7^ tons of kiln slag generated by other small smelteries, and 0.5 × 10^7^ tons of construction waste from Zhehai town. Moreover, the study area is not only affected by the slag heap but also by unknown historical pollution sources and stratum heterogeneity. Thus, it is difficult to prevent and control the groundwater pollution from the slag heap.

**Figure 1:**
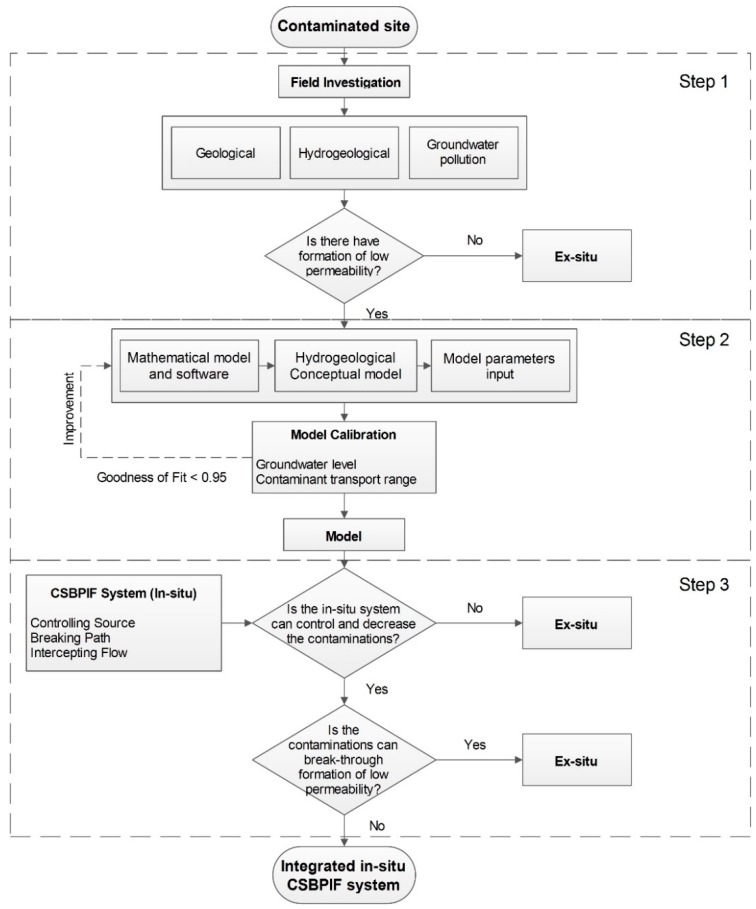
Flow chart for control of contaminant transport caused by open-air heavy metal slag.

This study focuses on the prevention of groundwater pollution from heavy metal leachate. First, the hydrogeological parameters of clay strata are obtained according to field surveys and laboratory experiments, and these parameters are used to create a hydrogeological conceptual model. Second, a numerical model is constructed to calculate the pollution range and degree of groundwater pollution associated with the open-air slag heap over 50 years. Third, the numerical model results are used to assess the feasibility of in situ treatment schemes to prevent groundwater pollution from the slag heap (Figure 1).

## 2. Background of the Study Area

### 2.1. Description of the Study Area

**Figure 2:**
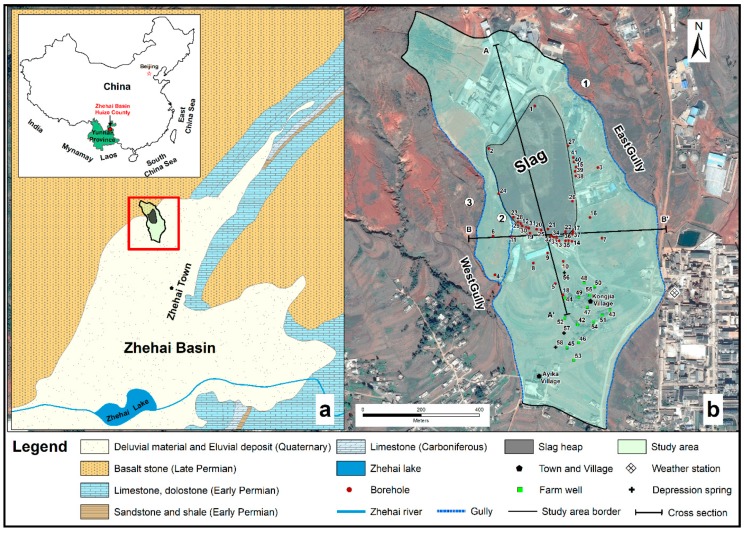
(**a**): Geological–hydrogeological map of the study area in the northwestern Zhehai Basin, northeastern Yunnan Province (China). (**b**): Locations map of the hydraulic head and groundwater quality observation points (boreholes, farm wells and depression springs). The study area border, the gullies (①-East gully, ② and ③-West gullies) and the research cross-sections (A–A’ and B–B’) is also located.

The slag heap is located in the northern Zhehai Basin in Yunnan Province (Figure 2). The study area is approximately 1.64 km^2^ in area and characterized by piedmont slopes and alluvial fan landforms. In addition, the ground elevation of the alluvial flood fan is between 2110 and 2140 m. To the north side of the area are weathered basalt hills. The southward transition to the Zhaihai Basin is relatively flat.

There are no perennial rivers or lakes near the slag heap. However, large gullies have developed on the east and west side of the heap, including the east gully ① and west gullies ② and ③, which formed in a north-south direction. The east gully is approximately 100–150 m from the slag heap. West gully ② is less than 5 m from the slag heap, and west gully ③ is 20–100 m from the slag heap. The deepest part of any given gully is 15 m deep. In addition, the gullies extend south downstream of the large slag pile. These gullies then converge with other gullies in the basin. The study area has a warm temperate plateau monsoon climate with a mean annual temperature of 12.7 °C and 792 mm of mean annual precipitation.

A detailed hydrogeological survey of the study area was conducted from 2014 to 2015. The groundwater level and quality were monitored at 58 observation points, including 41 boreholes, 14 farm wells near Kongjia Village and 3 depression springs on the south side of the slag heap. Among 41 boreholes, 5 (No. 3, 4, 5, 6 and 7) provided observations of both upper pore water and lower pore-fissure water.

### 2.2. Stratigraphic Configuration and Hydrogeological Characteristics

**Figure 3:**
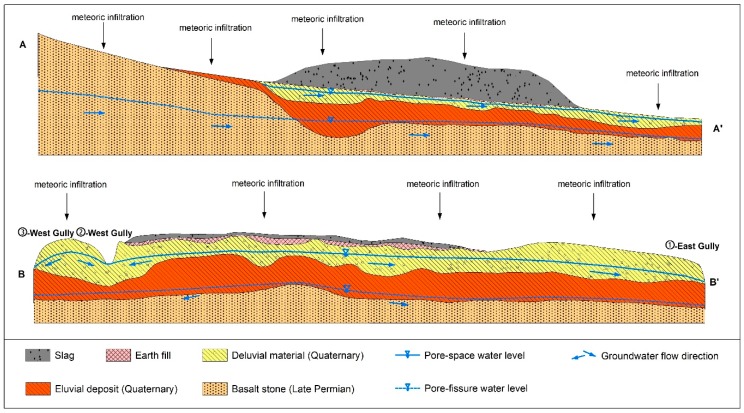
Cross-sections A–A’ (**A**) and B–B’ (**B**) of the geometrical-structural and two-aquifer groundwater system in the study area (Figure 2 shows cross-section locations).

The main stratigraphic configuration and hydrogeological characteristics of the aquifer system below the Zhehai slag heap were reconstructed according to the hydrogeologic map of Dongchuan, Yunnan Province and general geologic properties obtained from the 41 boreholes (Figure 3). The lithology includes three main groups with different permeabilities based on the local formations and results of a groundwater pumping test.

The upper layer, Deluvial Material (DM) of the Holocene and Upper Pleistocene, mainly consists of gravel and clay from slope materials and diluvium, with a thickness of 1–20 m. In addition, the hydraulic conductivity (K) ranges from 3.15 × 10^−8^–9.41 × 10^−6^ m/s and varies with changes in the percentage and size of the gravel in the formation.

The intermediate layer, which is an Eluvial Deposit (ED) of the Middle Pleistocene, consists of clay from slope materials and weathered residuals, with a continuous and stable spatial distribution and thickness varying from 10–30 m. In addition, the K ranges from 1.30 × 10^−10^–2.07 × 10^−8^ m/s.

The lowest layer is Permian Basalt Stone (BS) and is mainly composed of weathered or strongly weathered basalt, with a thickness varying from 40–130 m. In addition, the K is 6 × 10^−9^–2 × 10^−8^ cm/s.

A noncontinuous modern Earth Fills (EF) layer consisting of silty clay is located between the slag heap and the DM layer, with a thickness of 1–10 m and an average K of less than 1 × 10^−7^ cm/s. Moreover, the DM layer, ED layer and weathered BS layer contain silty clay, of which clay particles account for approximately 30% of each layer based on physical and chemical tests and a property analysis of the soil. Thus, these layers can effectively absorb heavy metals to some extent.

### 2.3. Piezometric Surface and Hydraulic Properties

The hydrogeological system in the study area can be divided into two low-permeability aquifers and two relative aquicludes. The upper low-permeability aquifer is the DM layer, and the ED layer forms the first aquiclude. The lower low-permeability aquifer and the relative aquiclude are composed of a completely weathered BS layer and a highly to moderately weathered BS layer.

**Figure 4:**
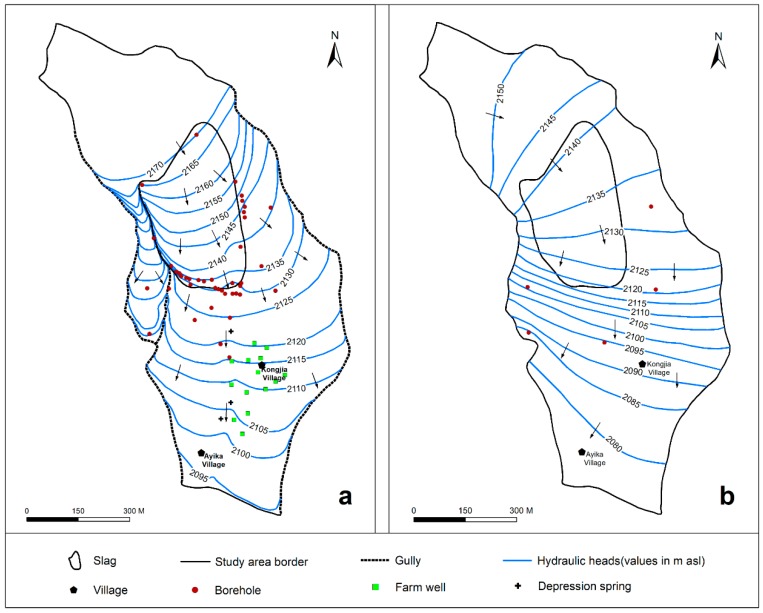
Groundwater level contour map in 2014: (**a**) is the water level contour map of pore water, and (**b**) is the water level contour map of lower pore-fissure water.

Figure 4 shows water level contour maps of the pore water (Figure 4a) in the upper part of the study area and the lower pore and fissure water (Figure 4b) in 2014. The main recharge mechanisms for the pore water are precipitation infiltration and lateral flow. In addition, the water depth is generally 2–15 m. On one hand, precipitation enters the pores through vertical infiltration from the surface. On the other hand, precipitation directly pollutes the pore water by transporting heavy metal leachate from the slag heap. Finally, the contaminated pore water flows along the hydraulic gradient to the south, i.e., downstream of the slag heap, and discharges into the gullies on the east and west sides of the heap.

The main recharge mechanism of the pore-fissure water is precipitation that flows along the stable weathered crust of the basalt into the BS aquifer in the hilly mountainous area. The direction of flow and discharge of pore-fissure water is mainly toward the center of the Zhehai Basin, with a water depth ranging from 10–30 m in this lower zone.

### 2.4. Groundwater and soil pollution

**Figure 5:**
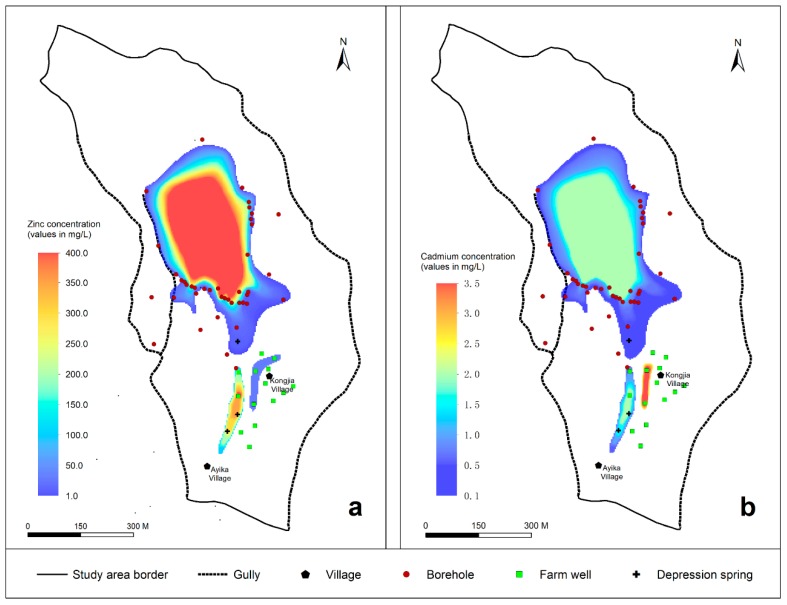
Concentrations of the characteristic pollutants in the pore water: (**a**) Zn and (**b**) Cd.

The groundwater pollution in the slag heap area from 2014 to 2015 showed that Zn (Figure 5a) and Cd (Figure 5b) were mainly concentrated in the pore water and that the pore-fissure water was not polluted. The distributions of the concentrations of Zn and Cd in the pore water were influenced by several factors in the groundwater environment.

Zn and Cd were mainly distributed in a circular area around the slag heap. The east and west sides of the circle are confined by the adjacent gullies (east side ① and west side ②). The east side ① and west side ② gullies work as natural barriers and prevent the leachate from migrating. Based on the direction of groundwater runoff, the groundwater contaminated by the slag heap mainly flows south and southeast from the slag heap.

The DM layer below the slag heap area is very heterogeneous. Complex groundwater channels exist in the DM aquifer, including some preferential flow paths. For example, the concentrations of Zn and Cd in the No. 5, 8, 9 and 10 boreholes were over 100 times greater than those at the No. 56 depression spring, No. 18 borehole and No. 44 farm well.

The area south of the slag heap is characterized by micro-geomorphological variations that influence contaminant transport, and the leaching from the slag heap and runoff are exchanged with surface water and groundwater to the south. For example, the Zn concentration in the No. 52 irrigation well reached 394 mg/L, which was higher than most of the other observations. This finding indicates that the leachate is exchanged with surface water and groundwater to the south of the slag heap and accumulates, mainly due to a slope decrease that impedes runoff, near the No. 52 irrigation farm well, resulting in higher concentrations of pollutants on the south side of the slag heap.

Some other pollution sources around Kongjia Village also pollute the area to the south of the slag heap to some degree. For example, the Cd concentrations in the No. 42 and No. 49 farm wells were not only much higher than those in the surrounding monitoring wells but also higher than the concentrations of leachate contaminants produced in the lower part of the slag heap.

Therefore, the causes of pollution in the study area are variable and result from the heterogeneous features (fast runoff channels) in the underlying strata, interactions between surface water and groundwater, and other historical pollution sources. Thus, an assessment of the study area and a numerical model of groundwater solute transport are required to accurately quantify the range and degree of the influence of the slag heap over a 50-year accumulation process. Based on the results, prevention and control schemes for groundwater pollution can be proposed.

## 3. Materials and Methods

### 3.1. Mathematical Model and Software

Based on the seepage equation and Darcy’s law, a three-dimensional (3D) mathematical model of unsteady flow in the simulated area was established [26]:

(1) $$\frac{\partial }{\partial x}\left(K_{x}\frac{\partial H}{\partial x}\right)+\frac{\partial }{\partial y}\left(K_{y}\frac{\partial H}{\partial y}\right)+\frac{\partial }{\partial z}\left(K_{z}\frac{\partial H}{\partial z}\right)+q_{s}=\mu_{s}\frac{\partial H}{\partial t}\;H\left(x,y,z,0\right)=H_{0}\left(x,y,z\right)\in \Omega \;K\frac{\partial H}{\partial n}|s_{2}=q\left(x,y,z,t\right)\left(x,y,z\right)\in S_{2}\;H\left(x,y,z,t\right)=H_{1}\left(x,y,z\right)\in S_{1}$$

where *Ω* is the volume of the flow model (*L^3^*), H is the hydraulic head (*L*), H_1_ is the head in the initial state, *S*_1_ is the 1st-type (Dirichlet) flow boundary condition, and *S*_2_ is the 2nd-type (Neumann) flow boundary condition. In addition, *K_x_*, *K_y_*, and *K_z_* are the hydraulic conductivities in the *x*, *y* and *z* directions (*L·T*^−1^); μ_s_ is the specific yield (dimensionless); *q*_s_ is the source/sink term (*L·T*^−1^); and *t* is the time (*T*).

The transport equation for a contaminant is based on the state of equilibrium adsorption, as follows [26]:

(2) $$R\frac{\partial C^{f}}{\partial t}=\frac{\partial }{\partial x}\left(D_{x}\frac{\partial C^{f}}{\partial x}\right)+\frac{\partial }{\partial y}\left(D_{y}\frac{\partial C^{f}}{\partial y}\right)+\frac{\partial }{\partial z}\left(D_{z}\frac{\partial C^{f}}{\partial z}\right)-v_{x}\frac{\partial C^{f}}{\partial x}-v_{y}\frac{\partial C^{f}}{\partial y}-v_{z}\frac{\partial C^{f}}{\partial z}$$

where *C^f^* is the concentration of dissolved species (*M·L*^−3^; mass per fluid volume); *R* is the retardation factor for the linear sorption isotherm (dimensionless); *v_x_*, *v_y_*, and *v_z_* are the seepage velocities in the *x*, *y* and *z* directions (*L·T*^−1^); and *D_x_*, *D_y_*, and *D_z_* are the hydrodynamic dispersion coefficients in the *x*, *y* and *z* directions (*L*^2^*·T*^−1^).

The retardation factor is computed by the following equation:

(3) $$R=1+\frac{K_{D}\rho_{b}}{\theta }$$

where *ρ_b_* is the bulk density (*M·L*^−3^), *θ* is the porosity (*dimensionless*), and *K_D_* is the distribution coefficient, which is the gradient of the linear sorption isotherm.

The flow and transport models are solved using Finite Element subsurface FLOW System (FEFLOW) software, which was developed by DHI-WASY (Berlin, Germany). FEFLOW is one of the most versatile software packages for simulating complex 3D unsteady groundwater flow and contaminant transport [27].

### 3.2. Model Conceptualization and Discretization

**Figure 6:**
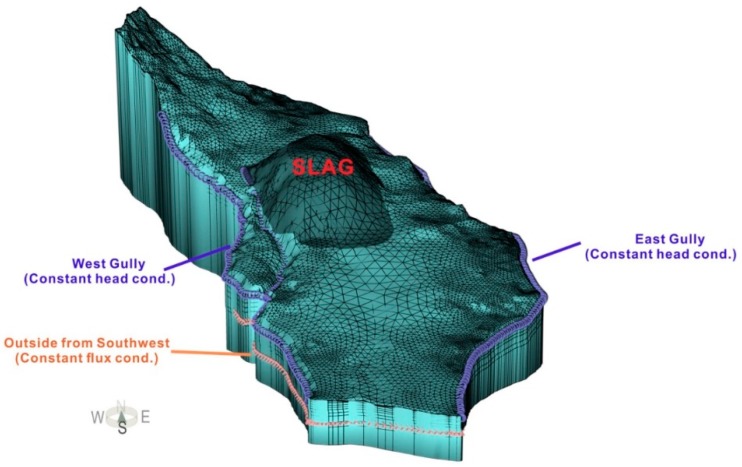
Three-dimensional(3D) numerical model and boundary conditions of the study area.

**Figure 7:**
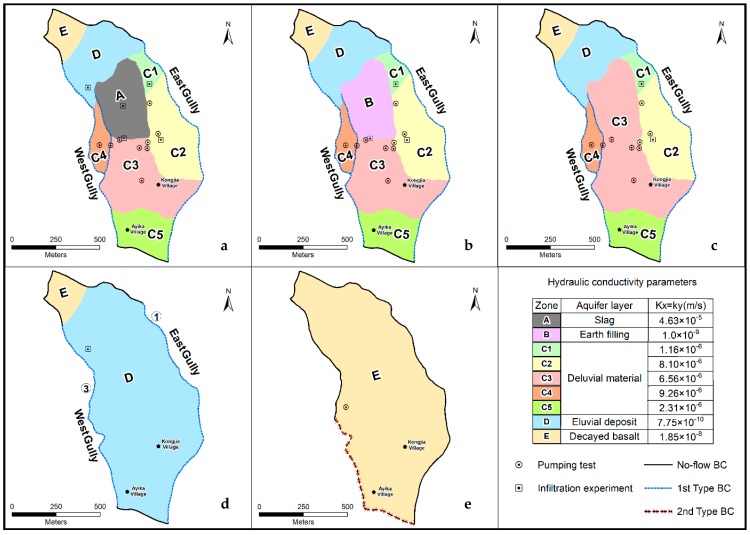
Hydraulic conductivity zone map: (**a**)-Slag heap layer (A), (**b**)-Earth filling layer (B), (**c**)-Deluvial material layer (C1, C2, C3, C4, and C5), (**d**)-Eluvial deposit layer (D), and (**e**)-Decayed basalt (E).

The study area is treated as a single hydrogeologic unit. The DM and BS aquifers form a heterogeneous, anisotropic and unsteady three-dimensional flow system (Figure 6). Figure 7 shows the strategy for setting the boundary conditions of the numerical model. The upper boundary of the model is a precipitation recharge and evaporation discharge boundary. The lower boundary of the model is an impermeable boundary based on the BS relative aquiclude. The northern part of the model is constrained by a zero-flux boundary. The west and east gullies form hydraulic head boundaries for the DM aquifer and zero-flux boundaries for the BS aquifer. On the southwest side of the slag heap, the boundary near Ayika village is a fixed flow boundary. Additionally, the southern boundary of the study area is a zero-flux boundary for the DM and BS aquifer.

A triangular mesh generation method was used to discretize the 3D models. Each grid slice contained 5596 nodes and 10,373 finite elements, with 78,344 nodes and 134,849 finite elements in the entire grid. A statistical analysis indicated that the number of obtuse angles greater than or equal to 90 degrees in the finite element mesh was 91 after dissection, or approximately 1% of all units. Thus, the mesh was effectively and accurately designed.

Moreover, to avoid numerical oscillations and dispersion in the model, auxiliary layers was established for vertical encryption. Therefore, the model consisted of 16 layers: the first through the fifth layers were slag heap layers, the sixth was the EF layer, the seventh through eleventh layers were the DM aquifer, the twelfth to fourteenth layers were the ED relative aquiclude, the fifteenth layer was the BS aquifer and the sixteenth layer was the BS relative aquiclude.

### 3.3. Model Input Parameters

Figure 7 shows that the ED layer and BS layer are exposed at the surface to the north of the slag heap. Additionally, to the east, west and south of the slag heap, the DM layer is exposed. In the vertical direction, the ED layer and BS layer are located below the DM layer.

A flow model is mainly affected by the hydraulic conductivity and specific yield, and the parameter values in this case were obtained based on a pumping experiment and double-ring infiltration experiment. Moreover, the boundary conditions, including the effects of precipitation-induced infiltration and the boundary values of the hydraulic head and fluid-flux boundaries of the model, are key factors in determining the model water balance. The steepness and depth of the large gullies on both sides of the slag heap made monitoring difficult. Hence, the model used east gully ① and west gullies ② and ③ as hydraulic head boundaries, and the required values were the model surface elevations. According to the observation data, Darcy flow boundary of the BS aquifer in the southwestern part of the study area is an outflow boundary, and fixed flow value is 4 × 10^−3^ m/d.

In the solute migration model, the active porosity and dispersity are extremely important, and these factors are highly influenced by the anisotropy of the study area. In addition, the clay can absorb heavy metal pollutants [28,29,30]. Accordingly, the solute transport model must include an appropriate adsorption equation, and the corresponding adsorption parameters should be obtained through indoor adsorption experiments.

The most common adsorption equations include the Langmuir equation and Freundlich adsorption equation. However, the Langmuir adsorption equation can better reflect high concentrations of subsurface pollutants [26] and is given as follows:

(4) Cs=m1Cf(1+m2Cf)

where *C*^s^ is the concentration of an absorbed species (*M·L*^−3^; mass per solid volume), *C^f^* is the concentration of a dissolved species (*M*·*L*^−3^; mass per fluid volume), m_1_ is a fitting parameter (unitless), and m_2_ is another fitting parameter (*L*^3^/*M*).

### 3.4. Flow Model Calibration

In a numerical model, the purpose of calibration is to reflect the actual hydrogeological conditions as well as possible. Steady and unsteady flow model corrections can be applied to calibrate a flow model. In the correction of the steady flow model, the hydraulic conductivity and specific yield were modified, and the simulated groundwater levels were fit to the measured levels at observation wells.

**Figure 8:**
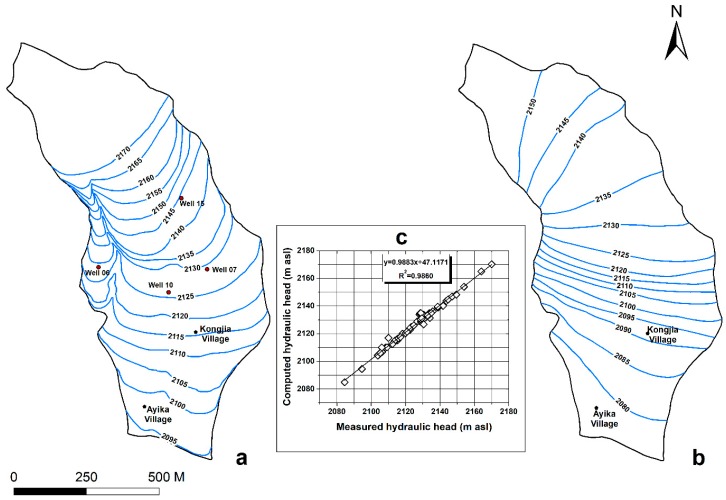
Model-simulated groundwater level contour maps and calibration curves under steady flow conditions: (**a**) Pore space groundwater level map, (**b**) Deep pore-fissure groundwater level map and (**c**) Calibration curves of the simulated and observed groundwater levels (comparison with 2014 values for 58 observation points).

Figure 8 illustrates the groundwater level isolines of the pore water in the steady flow model (Figure 8a) and deep pore-fissure water (Figure 8b). A comparison of the isolines of the measured groundwater level from June to September 2014 indicates that the fitting results are satisfactory. According to the measured and simulated groundwater levels at 41 boreholes (including the pore water of the DM aquifer and deep pore-fissure water of the BS aquifer) and 14 farm wells, the fitting results show (Figure 8c) that the average error between the measured groundwater level and simulated groundwater level at 55 observation holes is small. Thus, the accuracy of the steady flow model in the simulation area was verified.

**Figure 9:**
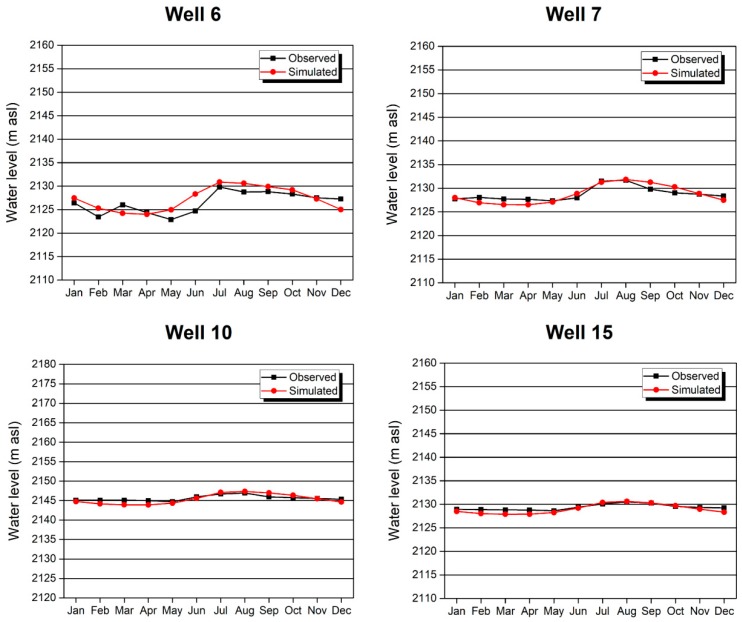
Comparison of the simulated groundwater level and measured groundwater level in different long-term observation wells under unsteady flow conditions (from January 2014 to January 2015).

The unsteady flow model simulates groundwater level changes over time. The groundwater level calculated by the steady flow model was used as the initial groundwater level in unsteady flow simulations, and groundwater level and infiltration time series were used in the calculations. After selecting annual average single-day monitoring data from 1981 to 2015 from the national meteorological stations and study area stations, we subtracted evapotranspiration from the data collected above. Considering the influences of the lithology and topographic slope on infiltration, we calculated the precipitation-based infiltration volume at the upper boundary of the model. Finally, we compared the groundwater levels changes and errors between the measured and calculated values based on long-term observations to calibrate and assess the fit of the unsteady flow model. Due to the focus on the migration of pollutants in the pore water of the DM aquifer layer, the long-term monitored groundwater levels in the No. 6, 7, 10 and 15 boreholes of the DM aquifer were available. As Figure 9 shows, from January 2014 to January 2015, in the No. 6, 7, 10 and 15 boreholes, the measured monthly groundwater levels based on long-term observations fit the model values very well.

Additionally, Figure 7 shows the value of model horizontal hydraulic conductivity after calibration. The vertical hydraulic conductivity is 1/10 of that in the horizontal direction. The reference empirical values of the specific yield are 26%, 5%, 2% and 0% for the slag heap, DM layer, ED layer, and BS layer, respectively.

## 4. Results and Discussion

### 4.1. Modeling Heavy Metal Pollutant Transport from the Tailings Pond

The Langmuir isotherm adsorption parameters m_1_ and m_2_ for Zn^2+^ and Cd^2+^ in different strata of the study area were calculated in an isothermal adsorption experiment (Table 1). Because of the continuous accumulation of smelting waste in the slag heap area over the past 50 years, the decay of the concentration of pollutants can be neglected. Consequently, the pollution source concentration *C*_0_(*t*) is treated as a constant (*C*_0_(*t*) = N, where *t* is time). According to the principle of risk maximization, the concentrations of Zn and Cd were set to the highest values from the groundwater pollution survey in 2014: *C*^Zn^_0_ = 400 mg/L, and *C*^Cd^_0_ = 1.5 mg/L. The solute assignment region is region A in Figure 7.

**Figure 10:**
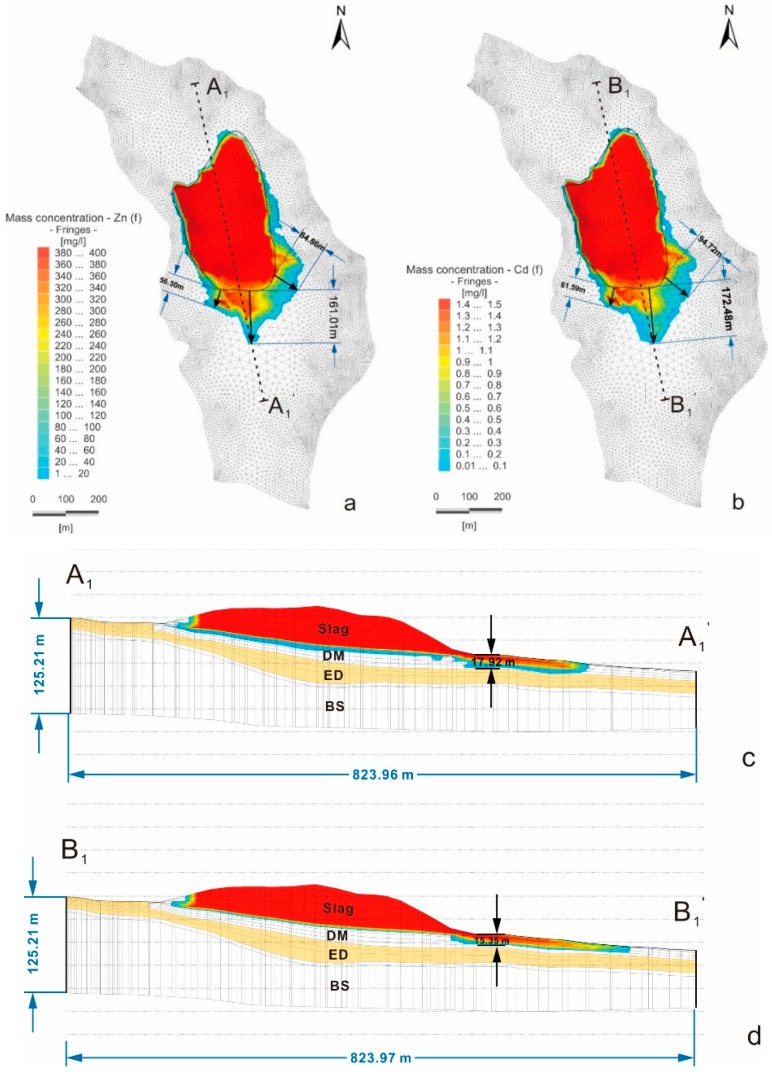
Model-simulated concentrations of the characteristic pollutants in the pore water: (**a**) Zn; (**b**) Cd; (**c**) Cross-section A_1_–A_1_’; and (**d**) Cross-section B_1_–B_1_’. DM: Deluvial Material, ED: Eluvial Deposit, BS: Basalt Stone.

The simulation result shows that Zn and Cd entered the pore water through atmospheric precipitation-induced infiltration through the slag heap from 1965 to 2014 (Figure 10). In the vertical direction, the maximum migration distance of Zn was 17.92 m (Figure 10c), and that of Cd was 15.35 m (Figure 10d), indicating that neither reached the lower pore-fissure region of the BS layer. In the pore water, the main runoff channels for pollutant transport flow south from the slag heap. Additionally, some flow southeast, a few flow southwest.

In the horizontal direction, the results demonstrate that Zn (Figure 10a) and Cd (Figure 10b) displayed the highest concentrations in the center of the slag heap, and a pollution halo distributed around the slag heap can be observed. This finding is supported by the pollutant concentration map (Figure 5) of measured values in the field, indicating that the fitting results of the contaminant transport model are satisfactory. In Figure 5, a statistical analysis of the circular distribution of the concentrations of Zn and Cd around the slag heap in the field showed that the maximum migration distance of Zn was 173 m to the south, 118 m to the southeast and 62 m to the southwest. Additionally, the maximum migration distance of Cd was 193 m to the south, 112 m to the southeast and 63 m to the southwest. In in Figure 10, the maximum migration distance of Zn is 161.01 m to the south, 84.86 m to the southeast and 56.30 m to the southwest. Additionally, the migration distances of Cd are 172.48 m to the south, 94.72 m to the southeast and 61.59 m to the southwest.

The results show that the heavy metal leachate pollutants have a limited migration distance in the pore water, with no spreading to the high pollution area near monitoring points 18, 44, 52, 57, and 58 on the south side of the slag heap, or to the southeast of Kongjia Village, as shown in Figure 5. Therefore, (1) the leachate pollutants produced by the slag heap exhibit limited migration and diffusion in groundwater toward the surrounding area, and (2) the groundwater pollution in the study area is not fully caused by the single slag heap pollution source.

In addition, the active porosity of each model layer was determined by inverse modeling. The active porosity values based on the method of Heath [31] were 0.25, 0.5, 0.7, and 0.6 for the slag heap, DM layer, ED layer, and BS layer, respectively. Additionally, the value of dispersion is affected by the macroscopic heterogeneity of the study area. Within a given scope, the dispersion increases with the scale of the field research site. Considering the dispersion degree and the reliability of the classification of the relevant observation scales [32], the longitudinal dispersion of the model is 1 m, and the lateral dispersion is 0.1 m.

### 4.2. Water Budget for the Slag Heap

Although the leachate pollutants have a limited impact on the study area, the area around the slag heap, especially the south side, is obviously affected due to the local micro-geomorphology. In addition, the groundwater contaminated in the runoff process to the south of the heap flows to the surface and migrates downstream with surface water. Therefore, the model could be used to quantitatively analyze the groundwater recharge, runoff and discharge processes around the slag heap to design an in situ treatment scheme.

**Figure 11:**
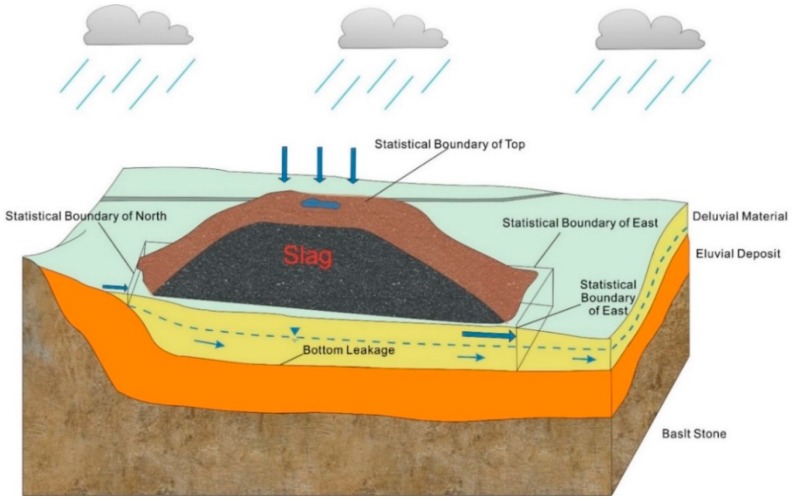
Schematic diagram showing the area of the numerical model consisting of six parts: precipitation-induced infiltration and pore water flows to the north, east, west, south and bottom of the DM aquifer.

**Figure 12:**
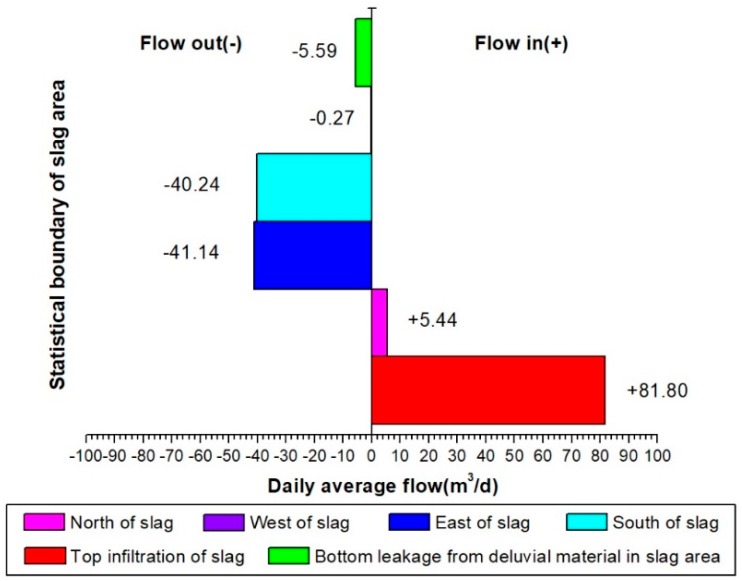
Daily average flow map for each area of the model.

Figure 11 shows areas of groundwater recharge, runoff and discharge flow around the slag heap in the model. Each area includes a total of 6 flow components: the infiltration flow from the top and the pore water flows in the DM aquifer to the north, east, west, south and bottom of the model. The daily average flow statistics (Figure 12) reveal that the pore water is recharged by two sources: atmospheric precipitation at a rate of 81.80 m^3^/d and groundwater runoff at a rate of 5.44 m^3^/d. Of these sources, precipitation-based infiltration accounts for 93.76% of total infiltration. In addition, infiltration transports leachate from the slag heap into the pore water in the DM aquifer. Therefore, the amount of leachate produced is directly related to the infiltration rate. It is assumed that the concentrations of Zn and Cd in the leachate are constant at *C*^Zn^_0_ = 400 mg/L and *C*^Cd^_0_ = 1.5 mg/L; thus, the emission levels of Zn and Cd into the surrounding environment are *G*^Zn^ = 32.72 kg/d and *G*^Cd^ = 0.12 kg/d.

The pore water contaminated by heavy metal leachate in the study area is discharged by groundwater runoff to the south at a rate of 40.24 m^3^/d, to the east at 41.14 m^3^/d and to the west at 0.27 m^3^/d. Additionally, this water flows from the bottom of the DM aquifer below the slag heap at a leakage rate of 5.59 m^3^/d. Among these flows, those to the south and east sides of the slag heap are the most important outflow channels for contaminated groundwater, and they account for 93.25% of the total discharge.

**Figure 13:**
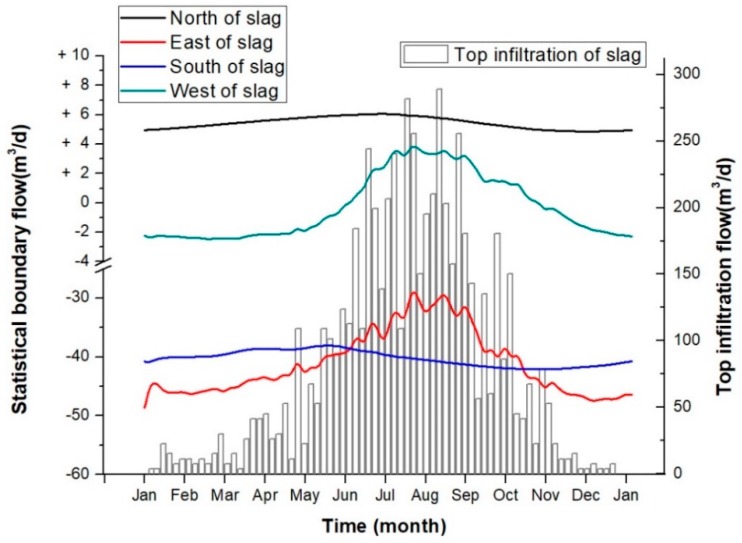
Dynamic flow plot of the areas in the model.

In Figure 13, the top infiltration value varies to a large extent due to the high hydraulic conductivity of the slag heap. The maximum value of single-day infiltration can reach 289.17 m^3^/d, and the minimum value is only 3.76 m^3^/d, a difference of 285.41 m^3^/d. During the high-water period from June to September, there is more precipitation-induced infiltration, and the daily average infiltration is 176.66 m^3^/d. During the relatively stable periods in May and October, the daily average infiltration is 59.30 m^3^/d. In the drought periods, which include January, February, November and December, the average daily infiltration is 15.62 m^3^/d. In addition, the groundwater flow in each area changes with the infiltration rate.

The north side of the heap is a recharge area, and the range of recharge variations is only 1.20 m^3^/d. The west side is a runoff exchange boundary, and the amplitude varies within 6.30 m^3^/d. During the stable and drought periods, the western side is a discharge boundary, and in the high-water period, it is a recharge boundary. However, the amplitudes of discharge and recharge are less than 4.0 m^3^/d. The south and east sides are where the main outflow occurs, and both are affected by precipitation-induced infiltration. However, a comparison of the flow curves between the south side and east side reveals a distinct trend. Notably, the outflow from the south side is more stable than that from the east side. The groundwater outflow from the south side minimally changes with infiltration, with an amplitude of only 4.03 m^3^/d, which is far less than the east side amplitude of 20.36 m^3^/d. Moreover, the flow from the southern boundary increases with the quantity of atmospheric precipitation and can be characterized as an outflow. During May, before the high-water period, the lowest outflow from the south side is 38.01 m^3^/d. In September, before the end of the high-water period, the maximum south side outflow can reach 42.04 m^3^/d. However, the outflow from the eastern boundary decreases with increasing infiltration. In July, during the high-water period, the lowest outflow from the east side is 28.31 m^3^/d, and in January, during the drought period, the highest outflow from the east side is 48.67 m^3^/d.

Therefore, the outflow from the south side boundary of the model area is relatively stable throughout the year and is minimally affected by changes in precipitation, and therefore infiltration. The southern boundary is the most important outflow boundary for the pore water below the slag heap. The outflow at the eastern boundary decreases with increasing infiltration and is a runoff exchange boundary. During the drought period, major outflow occurs at the eastern boundary. In the high-water period, the main flow type at the eastern boundary is discharge, even though runoff flows from this area to the other model areas. As supported by Figure 10, the leachate pollutants mainly migrate to the southeast of the slag heap, and some pollutants migrate from the northeast to the east side. As a result, the northeast side is a recharge boundary, and the southeast side is a drainage boundary.

### 4.3. Concerning an Integrated Pollution Control System

The in situ treatment of the slag heap should be based on an integrated system involving a “controlling the source, ‘breaking’ the path, and intercepting the flow” (CSBPIF) strategy. An ecological cover project could be adopted above the slag heap to reduce the amount of atmospheric precipitation that reaches the slag heap, thereby reducing the amount of leachate produced, which falls under “controlling the source”.

**Figure 14:**
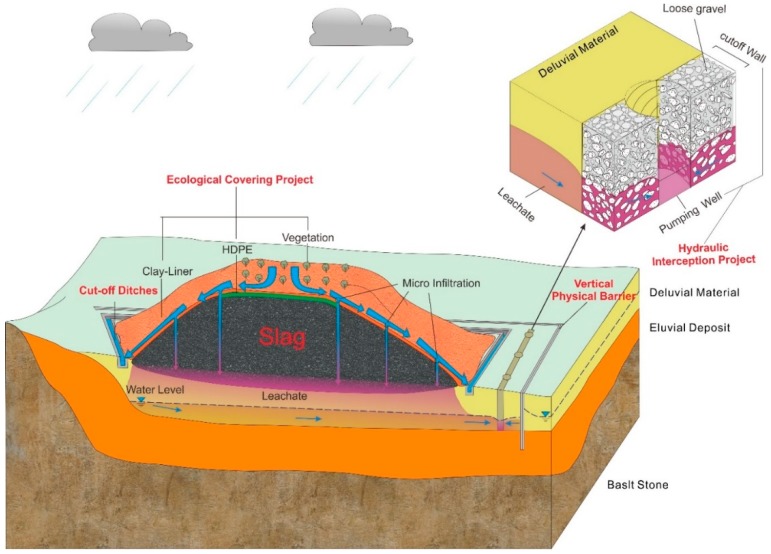
Diagram of the groundwater pollution prevention and control engineering strategy for the slag heap, including an ecological cover project for “controlling the source”, a vertical physical barrier for “‘breaking’ the path”, and a hydraulic interception project for “intercepting the flow”.

Physical measures for vertical partitioning in the lateral groundwater runoff zone of the slag heap to cut off flow channels could fulfill the “breaking the path” objective. Additionally, hydraulic interception projects could be implemented with the vertical partitioning measures to intercept and extract contaminated groundwater, thus preventing the spread of pollution. Such actions are classified as “intercepting flow” (Figure 14).

Specifically, the infiltration from the top of the slag heap produces the most leachate. The ecological cover project, which includes the installation of high-density polyethylene (HDPE), a clay liner and Vegetation, can reduce the amount of top infiltration and the quantity of sewage generated, thereby achieving the goal of controlling the groundwater pollution source. In addition, the major outflow of groundwater polluted by leachate occurs on the south side of the heap. The hydraulic interception project, which includes a cut off wall and vertical physical barrier arranged in the direction of vertical groundwater flow to the south, can effectively intercept and extract the polluted groundwater. This water is then purified at a sewage treatment plant to attain the goal of preventing polluted groundwater outflow. Groundwater runoff exchange occurs on the east side of the heap. Vertical physical barriers can be arranged to form a hydraulic barrier and reduce the lateral pore water recharge and discharge rates, thereby changing the groundwater flow direction from the east and cutting off the groundwater outflow channel to the south. In this scenario, the polluted groundwater flows to the south side and reaches a water treatment facility.

Moreover, the inflow from the north does not transport leachate, but it is still necessary to prevent surface water from entering the slag heap and producing leachate. Therefore, surface water cut off ditches can be created to intercept surface water. West gully ② limits the diffusion of groundwater to the peripheral area, and the rate of groundwater exchange is small. Thus, flow interception at west gully ② is sufficient.

### 4.4. In Situ Treatment Forecasting and Bottom Liner Design

The application of the CSBPIF system can prevent and control the groundwater pollution caused by the open-air slag heap. Moreover, the adsorption effect of clay can improve groundwater remediation in the cohesive soil area. Therefore, forecasting and comparative analyses are performed for two scenarios. Scenario A is based on natural circumstances, in which groundwater pollution situation associated with the slag heap occurs for 100 years without using the CSBPIF system. In scenario B, the groundwater polluted situation is modeled over 100 years using the CSBPIF system.

**Figure 15:**
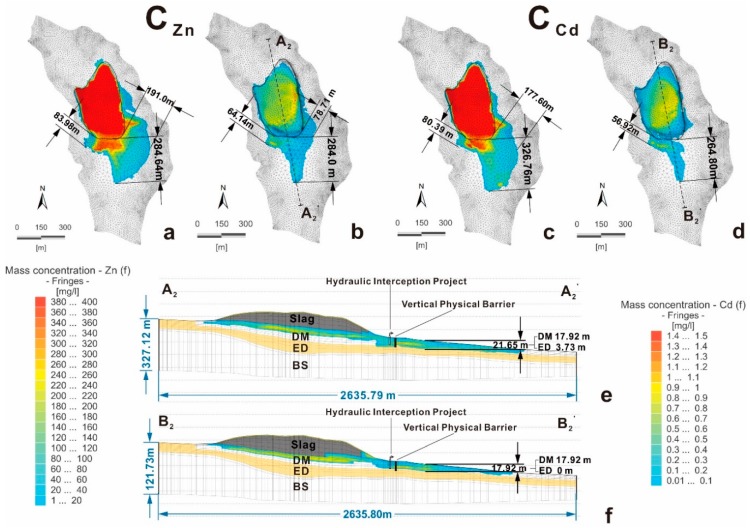
Model-forecasted concentrations of Zn and Cd in the pore water: (**a**) scenario A (Zn); (**b**) scenario B (Zn); (**c**) scenario A (Cd); (**d**) scenario B (Cd); (**e**) Cross-section A_2_–A_2_’; and (**f**) Cross-section B_2_–B_2_’.

Figure 15 shows the horizontal and vertical Zn and Cd distributions in the study area in scenarios A and B. Table 2 lists the migration and diffusion distances of Zn and Cd in scenarios A and B after 100 years (Figure 15), as well as the current values (Figure 10).

The result shows that the concentrations of Zn and Cd with the CSBPIF system for 100 years (Figure 15b,d) display a significant decrease compared to those under natural conditions (Figure 15a,c). Therefore, the CSBPIF system effectively lowers the concentrations of pollutants and cuts off the groundwater runoff channel from the slag heap to the surrounding area. However, the slag has already contaminated the pore water of the DM aquifer around the heap. As a result, the polluted groundwater outside the CSBPIF system still migrates and diffuses to the south.

To the southeast of slag, the maximum range of influence of Zn in scenario A decreased from the present value of 84.86 m to 78.71 m after 100 years. This value is half of the maximum range of 191.0 m observed in case B. The maximum range of influence of Cd changed from 94.72 m currently to 0.0 m in scenario A. These improvements indicate that the CSBPIF system not only directly reduces the pollution from the slag but can also restore the polluted groundwater quality to a certain extent due to the effect of cohesive soil adsorption.

Moreover, after 100 years, due to long-term slag pollution, there are relatively high concentrations of Zn and Cd to the southwest. The remediation of the groundwater quality requires more time, even though the CSBPIF system controls the pollution.

In addition, heavy metal pollutants travel only a small distance in vertical direction due to the retardation capacity of the clay strata and aquifers in the study area. Hence, the pollution prevention performance of the bottom liner layer of the CSBPIF system should be evaluated. In the study area, the DM aquifer and ED relative aquiclude both have low permeabilities and high adsorption capacities. Obviously, the hydraulic conductivity (K), retardation factor (R) and liner thickness L are the key parameters related to the performance of the liner layer.

Table 3 lists the minimum required thicknesses for the DM and ED when using the CSBPIF system in the study area. By changing the value of K, the numerical model can simulate the minimum thickness of the equivalent bottom liner in the CSBPIF system for Zn and Cd without considering the adsorption capacity. It is important to note that when the thickness of the liner layer is greater than 20 m and the corresponding K reaches 3.88 × 10^−8^ m/s, the impermeability decreases. The reasons for this decrease are not discussed in this work.

## 5. Conclusions

In this work, a conceptual model and a numerical model of groundwater contaminant transport in the study area are developed. The numerical model simulates the current pollution status of groundwater by the Zhehai slag heap over the 50-year period from 1965 to 2014. The simulation results show that the slag heap leachate only affects the pore water in the study area, and the concentrations of Zn and Cd are distributed in a circle surrounding the slag heap, mainly extending to the south and southeast. The concentrations are consistent with the field measurement results and support the accuracy and reliability of the model.

In this framework, the different flow areas around the slag heap are established in the numerical model. The flow statistics show that the rate of infiltration of atmospheric precipitation at the top of slag heap is 81.8 m^3^/d, accounting for 93.76% of total recharge. Additionally, the outflows to the south and east are 40.24 m^3^/d and 41.14 m^3^/d, respectively, accounting for 93.25% of the total discharge of contaminated pore water. Additionally, the south side of the study area is an absolute outflow boundary, and the east side is a runoff exchange boundary.

Consequently, a CSBPIF system could be used to stop further pollution and reduce the contamination of groundwater. The forecasting results using the CSBPIF system over 100 years indicate that the system can directly reduce the discharge of contaminants into the surrounding environment from the slag heap. The excellent adsorption effect of the clay strata in the study area can also remediate the polluted groundwater. To promote the application of the CSBPIF system, the equivalent bottom liner layers for DM and ED are obtain via model calculations.

Considering the environmental engineering investment, compared with ex situ schemes, the CSBPIF system can not only reduce the discharge of heavy metal pollutants into the groundwater environment to a greater extent but also reduce the engineering costs by tens of millions of dollars by avoiding landfill construction and waste residue transportation. In China, similar groundwater environment problems caused by industrial pollution are very severe. However, local governments and enterprises cannot afford the high costs of long-distance relocation and disposal projects. Therefore, the CSBPIF system provides an economic and effective solution.

## Figures and Tables

**Table 1:** Isothermal adsorption fitting parameters for Zn^2+^ and Cd^2+^.

Stratum	Zn			Cd		
	m_1_	m_2_	R	m_1_	m_2_	R
Deluvial material	66.0	0.004	28.340	2830.0	755.017	1.006
Eluvial deposit	49.0	0.003	21.250	2257.0	0.507	1457.430

**Table 2:** The migration and diffusion distances of Zn and Cd currently and after 100 years.

Elements	Scenarios	Horizontal Direction (m)			Vertical Direction (m)	
		South	Southeast	Southwest	DM Layer	ED Layer
Zn	Current situation	161.00	84.86	56.30	17.92	0.00
	Scenario A after 100 years	284.64	191.00	83.98	17.92	3.73
	Scenario B after 100 years	284.00	78.71	64.14	17.92	3.73
Cd	Current situation	172.48	94.72	61.59	15.35	0.00
	Scenario A after 100 years	326.76	177.60	80.39	17.92	0.00
	Scenario B after 100 years	264.80	0.00	56.92	17.92	0.00

**Table 3:** The thickness and pollution prevention performance of different bottom liner layers in the CSBPIF system.

Upper Bound of the Pollutant Concentration	Material Type	Material Name	Materia Properties		
			Vertical Hydraulic Conductivity (m/s)	Thickness (m)	Retardation Factor
*C*_Zn_ ≤ 400 mg/L	DM + ED	DM	6.56 × 10^−7^	<1.00 m	R ≥ 28.340
		ED	7.75 × 10^−11^	3.73	R ≥ 21.250
	Equivalent material	EM_Zn_-1 ^a^	7.75 × 10^−11^	10.37	R ≥ 1.0
		EM_Zn_-2	1.55 × 10^−10^	10.84	
		EM_Zn_-3	3.88 × 10^−10^	11.62	
		EM_Zn_-4	3.88 × 10^−9^	18.30	
		EM_Zn_-5	3.88 × 10^−8^	>20.00	
*C*_Cd_ ≤ 1.5 mg/L	DM + ED	DM	6.56 × 10^−7^	2.57	R ≥ 1.006
		ED	7.75 × 10^−11^	<1.00 m	R ≥ 1457.430
	Equivalent material	EM_Cd_-1	7.75 × 10^−11^	2.41	R ≥ 1.0
		EM_Cd_-2	1.55 × 10^−10^	2.73	
		EM_Cd_-3	3.88 × 10^−10^	3.84	
		EM_Cd_-4	3.88 × 10^−9^	11.83	
		EM_Cd_-5	3.88 × 10^−8^	>20.00	

^a^ EM is the abbreviation of the Equivalent material.
